# Patterns of electronic cigarette use and level of psychological distress

**DOI:** 10.1371/journal.pone.0173625

**Published:** 2017-03-09

**Authors:** Su Hyun Park, Lily Lee, Jenni A. Shearston, Michael Weitzman

**Affiliations:** 1 Department of Pediatrics, New York University School of Medicine, New York, New York, United States of America; 2 Department of Population Health, New York University School of Medicine, New York, New York, United States of America; 3 Brooklyn College, New York, New York, United States of America; 4 College of Global Public Health, New York University, New York, New York, United States of America; 5 New York University/ Abu Dhabi Public Health Research Center, Abu Dhabi, United Arab Emirates; 6 Department of Environmental Medicine, New York University, New York, New York, United States of America; Harvard Medical School, UNITED STATES

## Abstract

**Background:**

Psychological distress has been correlated with higher levels of nicotine dependence. To date, the possible association between individuals’ levels of psychological distress and e-cigarette use has not been investigated, despite the dramatic growth of e-cigarette use in the US. We examined this possible association using a nationally representative sample of US adults.

**Methods:**

A total of 36,697 adults from the 2014 National Health Interview Survey (NHIS) were included. The Kessler 6 scale was used to measure psychological distress. Multivariate logistic regression analysis was conducted to assess the association between level of psychological distress and e-cigarette use.

**Results:**

Both e-cigarette and cigarette use varied according to level of psychological distress as well as multiple socio-demographic characteristics. In a multivariate model, psychological distress was significantly associated with the following groups: (a) exclusive e-cigarette ever-use (aOR = 3.7; 95% CI = 1.6, 8.6), (b) current dual use of e-cigarettes and cigarettes (aOR = 4.6; 95% CI = 3.1, 6.7), (c) former cigarette use and ever use of e-cigarette (aOR = 3.2; 95% CI = 2.2, 4.8) and (d) current use of cigarettes only (aOR = 2.1; 95% CI = 1.7, 2.6).

**Conclusion:**

These are the first data to demonstrate that, as is true for cigarettes, e-cigarette use is associated with increased levels of psychological distress. Further large-scale, longitudinal studies are needed to determine the direction of this relationship and to evaluate the long-term positive and negative consequences of such use.

## Introduction

Tobacco use, largely in the form of cigarette smoking, remains the leading cause of death in the US and worldwide.[1] Cigarette use in the US has declined precipitously from 23% of adults and 29%[2] of adolescents in 2001 to 15% and 9%[3, 4], respectively, in 2015. In the last decade, however, novel alternative tobacco products and electronic nicotine delivery systems have entered the US market and have been met with overwhelming popularity by the general public as evidenced by their high rates of use among adults and adolescents. The widespread use of electronic cigarettes (e-cigarettes)[3, 5] has triggered a controversial public health debate regarding their potential effectiveness in smoking cessation versus their possible influence in the renormalization of cigarette smoking.[6–10]

Psychological distress which diminishes the quality of life and often is a central feature of many mental health problems is widely recognized as a crucial and pervasive public health concern [11, 12]. These problems are often associated with increased risk taking, including cigarette use and excessive alcohol consumption, and premature mortality.[13–15] Adults with mental health problems account for 31% of the cigarette use in the US and these individuals have significantly higher smoking rates, higher levels of nicotine dependence, and lower cessation rates than the general population.[16, 17] Furthermore, although smoking rates in the general population have declined over the past 50 years, smoking rates among those with mental health problems have remained high.[18–20]

Little, to date, is known about e-cigarette use among individuals with mental health problems. Two studies have found that smokers with severe mental health disorders, i.e. schizophrenia and bipolar disorder, especially those who wish to quit, may find e-cigarettes to be attractive substitutes for cigarettes[21, 22]. Only one study[23] that we are aware of has investigated the relationship between e-cigarette use and mental health utilizing a population based sample. In that study, mental health assessment consisted of yes-no responses to a specific question asking the respondent if they had one or more of the following: depression, anxiety disorder or other mental health condition. No scalar instrument was employed and there were no questions specifically about psychological distress.

In this paper, we report the results of analyses of a large, nationally representative sample of the US population to investigate whether the likelihood of e-cigarette use varies by levels of psychological distress. We used data from the 2014 National Health Interview Survey (NHIS). Psychological distress was measured in the NHIS using the Kessler 6 (K6) scale, which has been widely used as a screening instrument of individuals with possible mental health illness at the population level.[24, 25]

## Methods

### Study population

The NHIS is a cross-sectional nationally representative survey of the non-institutionalized US civilian population. The survey uses a complex, multistage, stratified probability design and is conducted annually by the National Center for Health Statistics (NCHS) of the Centers for Disease Control and Prevention (CDC). The Survey features face-to-face interviews that explore demographic characteristics and a broad range of health topics. A detailed description of the NHIS methodology is available online.[26] We analyzed data from the 2014 NHIS as this was the first year that it contained information on electronic cigarettes. The 2014 NHIS adult sample consisted of 36,697 respondents aged 18 years or older, and the response rate was 58.9%.[27] As the NHIS is publicly available, this study was exempt from institutional review board (IRB).

### E-cigarette and cigarette use

For the current study, we constructed five subgroups according to cigarette and e-cigarette use: those who ever used e-cigarettes exclusively, former cigarette users who ever used e-cigarettes, current dual users (cigarettes and e-cigarettes), those who currently use cigarettes exclusively, and individuals who never smoked cigarettes or e-cigarettes (non-users). Current e-cigarettes users (n = 81) and former e-cigarettes users (n = 547) were included in the “e-cigarettes ever users only” group (total n = 628). Separate analyses for those who were current exclusive users of e-cigarettes were not conducted because of the small sample size.

Ever use of e-cigarettes was defined by an answer of “yes” to the question “Have you ever used an e-cigarette, even one time?” “Exclusive e-cigarette ever-users” were defined as individuals who answered “yes” to the question about ever using an e-cigarette and “no” to the question “Have you smoked at least 100 cigarettes in your entire life?” “Ever e-cigarette users and former cigarette smokers” were defined as those who responded “yes” to the question about ever using an e-cigarette and had smoked at least 100 cigarettes in their lifetime but who had quit smoking at the time of interview. “Exclusive current cigarette users” were defined as those who had smoked 100 cigarettes in their lifetime, smoked “every day” or “on some days” at the time of the survey, and responded “no” to the question about ever using an e-cigarette. “Current dual users” included current cigarette users and those who answered “yes” to the question about ever using an e-cigarette and answered “every day” or “on some days” to the question “Do you now use e-cigarettes every day, some days, or not at all?” “Non-users” were defined as those who had never used either e-cigarettes or cigarettes.

### Psychological distress

The principal variable of interest, psychological distress, was measured using the Kessler 6 (K6) Scale, a six-item questionnaire that asks respondents whether they experienced the following feelings in the past 30 days: depression, nervousness, hopelessness, restlessness or fidgetiness, worthlessness, and/or that everything was an effort.[25] It is widely used as a non-specific mental health screening tool in the general population as noted by the Substance Abuse and Mental Health Services Administration (SAMHSA)[28] and others.[29] The K6 has been previously used in other studies to assess the association of mental health problems and tobacco use[30] as well as epilepsy, unhealthy sleep duration, abnormal body mass index and other chronic diseases.[31, 32] [33, 34]

Each item is scored on a five-point Likert scale from (0, none of the time; 1, a little of the time; 2, some of the time; 3 most of the time; and 4, all of the time), and the scores were summed to yield a total K6 score between 0 and 24. The Cronbach’s alpha for the sample was 0.86, reflecting a high level of internal consistency among the K6 items. As in other studies [32, 35], K6 scores were grouped into five levels of distress (0, 1 to 2, 3 to 5, 6 to 10, and ≥11) to explore potential dose- related effects in more detail. Approximately 50%, 20%, 15%, 10%, and 5% of the population, respectively, fell into these five categories.

### Covariates

Socio-demographic characteristics included age (18 to 24, 25 to 34, 35 to 44, 45 to 54, 55 to 64, and ≥ 65 years); race/ethnicity (non-Hispanic white, non-Hispanic black, Asian, Hispanic, and other); poverty ratio (<100% of the federal poverty line (FPL), 100–199% of the FPL, ≥200% of the FPL); education (less than high school, high school/graduate-equivalent degree, some college, and college/graduate degree); employment status in the last week; marital status (single, including never married, divorced, separated or widowed, and married); region of residence (Northeast, Midwest, South, and West); and health insurance coverage.

### Statistical analyses

Descriptive analyses were conducted on weighted percentages with 95% confidence intervals (CIs) to explore the distributions of socio-demographic factors and psychological distress levels in terms of e-cigarette and cigarette smoking status. Multivariate logistic regression was employed to seek independent associations between psychological distress levels and socio-demographic factors by e-cigarette and cigarette use status. A *p*-value less than 0.05 was regarded as statistically significant. To account for the complex sampling design, all analyses were performed using the survey module of Stata software version 14.0 (StataCorp, College Station, TX).

## Results

Table 1 presents the unweighted sample sizes and the weighted percentage distributions of selected socio-demographic characteristics and the psychological distress levels (K6) of the sample classified by e-cigarette and cigarette use status. Overall, the prevalence of exclusive e-cigarette ever-use was 3.2% (95% CI = 2.8, 3.6), of ever using e-cigarettes and formerly using cigarettes was 4.4% (95% CI = 4.0, 4.7), of current dual use was 4.3% (95% CI = 3.8, 4.8), of current cigarette-use only was 12.9% (95% CI = 12.3, 13.5), and the prevalence of non-use was 59.0% (95% CI = 58.3, 59.8) (Table 1). E-cigarette and cigarette use varied substantially by socio-demographic characteristics and levels of psychological distress.

**Table 1:** Selected characteristics by e-cigarette and cigarette use, 2014 National Health Interview Survey (n = 36,697)^a^.

	Total N^a^ (%^b^)	E-cigarette ever users only^c^ (n = 628) ^a^	Former cigarette users & e-cigarette ever users^d^ (n = 898) ^a^	Current dual users (cigarette and e-cigarette)^e^ (n = 935) ^a^	Current cigarette users only^f^ (n = 3,446) ^a^	Non-users (no cigarette or e-cigarette)^g^ (n = 21,196) ^a^
		% (95%CI) ^b^	% (95%CI) ^b^	% (95%CI) ^b^	% (95%CI) ^b^	% (95%CI) ^b^
**Psychological distress (K6)**^h^						
0	16,921 (48.9)	2.1 (1.7, 2.5)	2.6 (2.3, 3.1)	2.5 (2.1, 2.9)	11.0 (10.3, 11.8)	64.1 (63.1, 65.1)
1–2	7,121 (20.2)	3.3 (2.7, 4.2)	5.2 (4.3, 6.2)	4.6 (3.2, 6.6)	11.4 (10.2, 12.8)	59.3 (57.6, 60.9)
3–5	5,514 (15.3)	4.7 (3.8, 5.9)	6.4 (5.2, 7.7)	4.9 (3.9, 6.1)	14.3 (12.8, 15.9)	55.7 (54.0, 57.4)
6–10	3,807 (10.5)	6.0 (4.5, 8.0)	7.9 (6.1, 10.1)	9.4 (6.7, 13.2)	18.6 (15.5, 22.1)	50.6 (47.9, 53.2)
11–24	2,027 (5.1)	6.8 (3.7, 12.4)	7.1 (5.3, 9.5)	12.9 (10.4, 16.0)	25.6 (22.4, 29.1)	40.8 (37.7, 44.1)
**Gender**						
Female	20,299 (51.0)	1.5 (1.2, 1.8)	2.4 (2.1, 2.7)	2.5 (2.1, 3.0)	7.5 (6.9, 8.1)	64.6 (63.5, 65.6)
Male	16,398 (47.5)	2.5 (2.1, 2.9)	3.0 (2.6, 3.4)	2.8 (2.3, 3.3)	10.0 (9.4, 10.7)	53.1 (52.1, 54.1)
**Age**						
18 to 24	3,353 (12.6)	7.5 (6.2, 9.0)	3.7 (2.9, 4.8)	3.3 (2.1, 5.3)	6.3 (4.7, 8.3)	69.4 (66.7, 72.0)
25 to 34	6,431 (17.5)	3.6 (3.0, 4.4)	4.6 (3.9, 5.5)	3.5 (2.9, 4.3)	9.0 (8.1, 9.9)	61.4 (59.7, 63.1)
35 to 44	5,947 (16.6)	0.8 (0.5, 1.0)	3.3 (2.8, 4.0)	2.9 (2.3, 3.6)	10.2 (9.2, 11.3)	62.4 (60.7, 64.1)
45 to 54	6,117 (17.9)	1.0 (0.6, 1.7)	1.7 (1.4, 2.1)	3.1 (2.2, 4.3)	10.7 (9.7, 11.8)	59.4 (57.5, 61.3)
55 to 64	6,205 (16.6)	0.3 (0.2, 0.4)	2.1 (1.7, 2.7)	2.3 (1.8, 2.9)	10.1 (9.1, 11.2)	52.6 (50.9, 54.3)
65+	8,644 (18.8)	0.1 (0.0, 0.2)	0.9 (0.7, 1.3)	1.0 (0.8, 1.3)	5.7 (5.1, 6.5)	52.2 (50.7, 53.7)
**Race/ethnicity**						
NH white	22,779 (65.7)	1.9 (1.6, 2.2)	3.3 (3.0, 3.6)	3.3 (2.9, 3.8)	8.6 (8.0, 9.2)	53.7 (52.8, 54.7)
NH black	4,896 (11.6)	1.2 (0.9, 1.5)	1.1 (0.8, 1.6)	1.2 (0.8, 1.6)	12.6 (11.4, 13.8)	66.5 (64.5, 68.3)
Asian	2,025 (5.5)	1.9 (1.3, 2.7)	1.3 (0.8, 2.1)	0.9 (0.5, 1.6)	6.4 (5.0, 8.2)	76.7 (74.1, 79.2)
Hispanic	6,053 (15.3)	2.8 (2.2, 3.7)	1.6 (1.2, 2.0)	1.3 (0.9, 1.7)	6.9 (6.2, 7.7)	70.8 (69.2, 72.4)
Other	944 (2.0)	2.8 (1.7, 4.7)	3.4 (2.2, 5.1)	4.6 (2.6, 8.2)	12.0 (9.1, 15.6)	51.6 (46.3, 56.8)
**Poverty ratio**						
<100% FPL	6,150 (13.9)	2.3 (1.7, 2.9)	2.0 (1.6, 2.4)	3.7 (3.0, 4.5)	14.9 (13.6, 16.4)	55.4 (53.6, 57.2)
100–199% FPL	7,315 (18.9)	1.7 (1.4, 2.2)	2.2 (1.8, 2.6)	3.4 (2.9, 4.1)	11.5 (10.5, 12.6)	55.5 (53.9, 57.2)
≥ 200% FPL	21,302 (67.3)	2.1 (1.8, 2.4)	3.1 (2.7, 3.4)	2.2 (1.8, 2.6)	6.6 (6.1, 7.2)	60.6 (59.7, 63.6)
**Education**						
Less than HS	3,688 (7.1)	0.7 (0.4, 1.2)	1.2 (0.9, 1.8)	1.8 (1.4, 2.5)	16.5 (15.0, 18.2)	53.5 (51.2, 55.7)
HS/GED	7,962 (19.7)	1.6 (1.2, 2.1)	2.6 (2.1, 3.1)	3.5 (2.7, 4.6)	14.1 (12.9, 15.3)	49.9 (48.3, 51.6)
Some college	11,932 (32.7)	2.7 (2.3, 3.3)	3.3 (2.9, 3.8)	3.8 (3.2, 4.5)	9.5 (8.7, 10.5)	54.1 (52.8, 55.4)
BA or more	13,041 (40.6)	1.7 (1.4, 2.1)	2.5 (2.1, 2.9)	1.4 (1.2, 1.8)	4.1 (3.7, 4.6)	68.3 (67.3, 69.3)
**Employment status**						
Employed	21,420 (61.2)	2.4 (2.1, 2.8)	3.0 (2.7, 3.4)	2.6 (2.3, 3.0)	8.1 (7.6, 8.7)	61.8 (60.9, 62.6)
Unemployed	15,218 (38.8)	1.2 (1.0, 1.5)	2.1 (1.8, 2.4)	2.7 (2.2, 3.3)	9.6 (9.0, 10.4)	54.7 (53.6, 55.9)
**Marital status**						
Married	18,301 (60.4)	1.3 (1.0, 1.5)	2.6 (2.3, 2.9)	2.4 (2.0, 2.9)	7.5 (7.0, 8.1)	59.4 (58.4, 60.4)
Single	18,310 (39.7)	3.0 (2.6, 3.5)	2.8 (2.5, 3.2)	3.0 (2.7, 3.5)	10.6 (9.8, 11.3)	58.5 (57.4, 59.5)
**Region**						
Northeast	5,919 (17.3)	1.1 (0.8, 1.6)	2.0 (1.5, 2.7)	1.8 (1.4, 2.5)	8.6 (7.7, 9.6)	59.8 (58.3, 61.4)
Midwest	7,809 (23.0)	2.3 (1.7, 2.9)	2.6 (2.2, 3.2)	3.4 (2.6, 4.4)	10.4 (9.2,11.8)	54.5 (52.7, 56.2)
South	12,896 (37.2)	1.6 (1.3, 2.0)	2.8 (2.4, 3.3)	2.7 (2.2, 3.3)	9.2 (8.6, 9.9)	59.2 (58.0, 60.4)
West	10,073 (22.5)	2.9 (2.4, 3.5)	3.0 (2.6, 3.4)	2.4 (2.0, 2.9)	6.2 (5.5, 6.9)	62.8 (61.4, 64.1)
**Health Insurance**						
Yes	34,112 (94.4)	1.9 (1.7, 2.2)	2.6 (2.4, 2.9)	2.5 (2.2, 2.9)	8.4 (7.9, 8.9)	59.3 (58.5, 60.1)
No	2,516 (5.6)	2.0 (1.5, 2.7)	3.2 (2.4, 4.2)	4.3 (3.4, 5.5)	14.6 (12.8, 16.7)	53.5 (50.6, 56.3)

CI = confidence intervals; NH = non-Hispanic; K6 = Kessler 6;FPL = federal poverty line

^a^Unwerighted sample sizes

^b^Percentages are weighted (95% Confidence Interval)

^c^E-cigarette ever users only were defined as individuals who answered “yes” to the question about ever using an e-cigarette and “no” to the question “Have you smoked at least 100 cigarettes in your entire life?”

^d^Former cigarette users & e-cigarette ever users were defined as those who responded “yes” to the question about ever using an e-cigarette and had smoked at least 100 cigarettes in their lifetime but who had quit smoking at the time of interview.

^e^Current dual users included current cigarette users and those who answered “yes” to the question about ever using an e-cigarette and answered “every day” or “on some days” to the question “Do you now use e-cigarettes every day, some days, or not at all?”

^f^Current cigarette users only were defined as those who had smoked 100 cigarettes in their lifetime, smoked “every day” or “on some days” at the time of the survey, and responded “no” to the question about ever using an e-cigarette.

^g^Non-users were defined as those who had never used either e-cigarettes or cigarettes.

^h^K6 scores were grouped into five levels of distress (0, 1 to 2, 3 to 5, 6 to 10, and ≥11)

Females were less likely to use cigarettes and e-cigarettes than were males (1.5% vs. 2.5% for exclusive e-cigarette ever use). Unlike the case for cigarette use, younger adults (ages 18–24 years) were more likely to use e-cigarettes than were older adults. The percentages of the current dual users and the former cigarette smokers who ever used e-cigarettes were highest among those aged 25 to 34 years (3.5%, 4.6%, respectively). Current cigarette-use only was highest among non-Hispanic blacks (12.6%) and lowest among Asians (6.4%). In contrast, exclusive e-cigarettes ever use was lowest among non-Hispanic blacks (1.2%). For all categories of those who used e-cigarettes and/or cigarettes, rates of use increased with increasing levels of psychological distress, whereas the converse was true among those who used neither cigarettes nor e-cigarettes (Table 1).

Table 2 shows the results of the multivariate logistic regression used to assess independent relationships between levels of psychological distress and e-cigarette and cigarette use status. After adjustment for covariates, respondents with higher levels of psychological distress were at increased risk of being users versus non-users of e-cigarettes and cigarettes. Higher psychological distress levels were associated with a greater likelihood of exclusive e-cigarette ever-use (aOR = 3.7; 95% CI = 1.6, 8.6), ever-e-cigarette use and former cigarette use (aOR = 3.2; 95% CI = 2.2, 4.8), current dual use (aOR = 4.6; 95% CI = 3.1, 6.7), and exclusive current cigarette use (aOR = 2.1, 95% CI = 1.7, 2.6). The odds ratios for e-cigarette and/or cigarette use rose in an approximately linear manner with the level of psychological distress.

**Table 2:** Multivariate logistic regression model (aOR^a^, 95% CI) for the association between smoking status and psychological distress, and covariates, NHIS 2014.

	E-cigarette ever users only^b^ vs. non-users	Former cigarette users & e-cigarette ever users^c^ vs. non-users	Current dual users^d^ (cigarette and e-cigarette) vs. non-users	Current cigarette users only^e^ vs. non-users	Non-users (no cigarette or e-cigarette)^f^ vs. any-users
	aOR (95%CI)	aOR (95%CI)	aOR (95%CI)	aOR (95%CI)	aOR (95%CI)
**Psychological distress (K6)**^g^					
0	Referent	Referent	Referent	Referent	Referent
1–2	1.5 (1.1, 2.0)*	1.9 (1.5, 2.5)**	1.8 (1.1, 2.8)*	1.1 (1.0, 1.3)	0.8 (0.7, 0.9)**
3–5	2.3 (1.7, 3.3)**	2.5 (1.9, 3.3)**	1.8 (1.3, 2.5)*	1.4 (1.2, 1.6)**	0.7 (0.6, 0.7)**
6–10	2.8 (1.8, 4.2)**	2.9 (2.1, 4.0)**	3.0 (2.2, 4.1)**	1.7 (1.3, 2.2)**	0.5 (0.5, 0.6)**
11–24	3.7 (1.6, 8.6)*	3.2 (2.2, 4.8)**	4.6 (3.1, 6.7)**	2.1 (1.7, 2.6)**	0.4 (0.3, 0.5)**
**Gender**					
Female	Referent	Referent	Referent	Referent	Referent
Male	1.8 (1.4, 2.4)**	1.7 (1.4, 2.0)**	1.6 (1.3, 2.1)**	2.0 (1.7, 2.3)**	0.5 (0.5, 0.6)**
**Age**					
18 to 24	8.6 (5.5, 13.5)**	0.9 (0.6, 1.3)	0.5 (0.3, 0.7)**	0.3 (0.2, 0.5)**	1.8 (1.5, 2.1)**
25 to 34	5.0 (3.4, 7.5)**	1.4 (1.1, 1.8)*	1.1 (0.8, 1.5)	0.8 (0.7, 1.0)*	1.0 (0.9, 1.1)
35 to 44	Referent	Referent	Referent	Referent	Referent
45 to 54	1.5 (0.8, 2.6)	0.5 (0.3, 0.6)**	0.9 (0.6, 1.4)	0.9 (0.8, 1.1)	1.0 (0.9, 1.1)
55 to 64	0.5 (0.3, 0.9)*	0.6 (0.4, 0.8)*	0.7 (0.5, 1.0)*	0.9 (0.8, 1.1)	0.8 (0.7, 0.8)**
65+	0.1 (0.0, 0.3)**	0.2 (0.2, 0.4)**	0.3 (0.2, 0.4)**	0.4 (0.3, 0.5)**	0.8 (0.7, 0.9)**
**Race/ethnicity**					
NH white	Referent	Referent	Referent	Referent	Referent
NH black	0.4 (0.3, 0.6)**	0.2 (0.1, 0.3)**	0.2 (0.1, 0.3)**	0.7 (0.6, 0.9)**	2.0 (1.8, 2.2)**
Asian	0.8 (0.5, 1.3)	0.3 (0.2, 0.5)**	0.3 (0.1, 0.5)**	0.7 (0.5, 0.9)*	2.2 (1.9, 2.5)**
Hispanic	0.8 (0.6, 1.1)	0.2 (0.2, 0.3)**	0.2 (0.1, 0.3)**	0.3 (0.3, 0.4)**	2.6 (2.3, 2.8)**
Other	0.8 (0.4, 1.5)	0.8 (0.5, 1.3)	1.0 (0.5, 1.9)	1.4 (1.0, 2.0)*	0.9 (0.7, 1.1)
**Poverty ratio**					
<100% FPL	Referent	Referent	Referent	Referent	Referent
100–199% FPL	1.0 (0.7, 1.4)	1.2 (0.9, 1.6)	0.9 (0.7, 1.2)	0.8 (0.7, 1.0)	1.1 (1.0, 1.2)
≥ 200% FPL	1.5 (1.0, 2.2)*	1.6 (1.1, 2.2)*	0.6 (0.4, 0.8)*	0.6 (0.5, 0.8)**	1.2 (1.0, 1.3)*
**Education**					
Less than HS	Referent	Referent	Referent	Referent	Referent
HS/GED	1.6 (0.8, 3.3)	1.3 (0.8, 2.1)	1.4 (0.9, 2.1)	0.8 (0.7, 1.0)*	1.0 (0.9, 1.1)
Some college	2.0 (1.0, 3.8)*	1.2 (0.8, 2.0)	1.3 (0.9, 2.0)	0.5 (0.4, 0.6)**	1.1 (1.0, 1.2)
BA or more	1.0 (0.5, 2.0)	0.6 (0.3, 0.9)*	0.4 (0.3, 0.7)**	0.2 (0.1, 0.2)**	2.2 (1.9, 2.5)**
**Employment status**					
Employed	Referent	Referent	Referent	Referent	Referent
Unemployed	0.8 (0.6, 1.0)	1.1 (0.9, 1.4)	1.0 (0.8, 1.3)	1.2 (1.1, 1.4)*	0.9 (0.8, 1.0)*
**Marital status**					
Married	Referent	Referent	Referent	Referent	Referent
Single	1.2 (0.9, 1.6)	1.0 (0.8, 1.3)	1.3 (1.0, 1.6)*	1.3 (1.2, 1.5)**	0.9 (0.9, 1.0)
**Region**					
Northeast	Referent	Referent	Referent	Referent	Referent
Midwest	2.0 (1.2, 3.4)*	1.1 (0.8, 1.6)	1.4 (1.0, 2.1)	1.2 (1.0, 1.5)	0.9 (0.8, 1.0)
South	1.6 (1.0, 2.7)*	1.4 (1.0, 2.0)	1.5 (1.0, 2.2)*	1.0 (0.8, 1.2)	1.0 (0.9, 1.1)
West	2.3 (1.4, 3.7)*	1.3 (1.0, 1.9)	1.2 (0.8, 1.8)	0.7 (0.6, 0.9)*	1.1 (1.0, 1.2)
**Health Insurance**					
Yes	Referent	Referent	Referent	Referent	Referent
No	0.9 (0.6, 1.3)	1.3 (0.9, 1.8)	1.3 (1.0, 1.8)	1.3 (1.0, 1.6)*	0.8 (0.7, 1.0)*

^a^Adjusted for age, gender, race/ethnicity, poverty ratio, educational level, employment status, marital status, region and health insurance

^b^E-cigarette ever users only were defined as individuals who answered “yes” to the question about ever using an e-cigarette and “no” to the question “Have you smoked at least 100 cigarettes in your entire life?”

^c^Former cigarette users & e-cigarette ever users were defined as those who responded “yes” to the question about ever using an e-cigarette and had smoked at least 100 cigarettes in their lifetime but who had quit smoking at the time of interview.

^d^Current dual users included current cigarette users and those who answered “yes” to the question about ever using an e-cigarette and answered “every day” or “on some days” to the question “Do you now use e-cigarettes every day, some days, or not at all?”

^e^Current cigarette users only were defined as those who had smoked 100 cigarettes in their lifetime, smoked “every day” or “on some days” at the time of the survey, and responded “no” to the question about ever using an e-cigarette.

^f^Non-users were defined as those who had never used either e-cigarettes or cigarettes.

^g^K6 scores were grouped into five levels of distress (0, 1 to 2, 3 to 5, 6 to 10, and ≥11)

CI = confidence intervals; NH = non-Hispanic; K6 = Kessler 6;aOR = adjusted odds ratio; FPL = federal poverty line

*p<0.05

**p<0.001

Table 3 shows the results of a multivariate logistic regression that included all of the covariates in Table 2 but was restricted to individuals aged 18–34 years as these were the individuals most likely to have used cigarettes and e-cigarettes. It demonstrated essentially the same findings as those found for the entire adult sample shown in Table 2, i.e. higher levels of psychological distress were independently associated with increased use of both e-cigarettes and cigarettes in a fashion that suggested a dose response relationship, even among those who had used e-cigarettes but smoked less than 100 cigarettes.

**Table 3:** Multivariate logistic regression model (aOR^a^, 95% CI) for the association between smoking status and psychological distress, and covariates among individuals aged 18–34, NHIS (n = 9,784).

	E-cigarette ever users only^b^ vs. non-users	Former cigarette users & e-cigarette ever users^c^ vs. non-users	Current dual users (cigarette and e-cigarette)^d^ vs. non-users	Current cigarette users only^e^ vs. non-users	Non-users (no cigarette or e-cigarette)^f^ vs. any-users
Psychological distress (K6)^g^	aOR (95%CI)	aOR (95%CI)	aOR (95%CI)	aOR (95%CI)	aOR (95%CI)
0	Referent	Referent	Referent	Referent	Referent
1–2	1.4 (1.0, 2.1)	2.2 (1.5, 3.4)**	1.5 (0.9, 2.5)	0.9 (0.7, 1.3)	0.8 (0.6, 0.9)*
3–5	2.3 (1.6, 3.4)**	3.0 (1.9, 4.9)**	1.7 (1.0, 2.9)	1.3 (0.9, 1.8)	0.5 (0.4, 0.6)**
6–10	2.1 (1.3, 3.2)*	3.2 (1.9, 5.4)**	2.5 (1.5, 4.2)**	1.9 (1.1, 3.4)*	0.5 (0.4, 0.6)**
11–24	3.5 (1.2, 9.7)*	3.2 (1.7, 6.1)**	5.0 (2.8, 8.9)**	2.5 (1.6, 3.8)**	0.3 (0.2, 0.4)**

^a^Adjusted for gender, race/ethnicity, poverty ratio, educational level, employment status, marital status, region and health insurance

^b^E-cigarette ever users only were defined as individuals who answered “yes” to the question about ever using an e-cigarette and “no” to the question “Have you smoked at least 100 cigarettes in your entire life?”

^c^Former cigarette users & e-cigarette ever users were defined as those who responded “yes” to the question about ever using an e-cigarette and had smoked at least 100 cigarettes in their lifetime but who had quit smoking at the time of interview.

^d^Current dual users included current cigarette users and those who answered “yes” to the question about ever using an e-cigarette and answered “every day” or “on some days” to the question “Do you now use e-cigarettes every day, some days, or not at all?”

^e^Current cigarette users only were defined as those who had smoked 100 cigarettes in their lifetime, smoked “every day” or “on some days” at the time of the survey, and responded “no” to the question about ever using an e-cigarette.

^f^Non-users were defined as those who had never used either e-cigarettes or cigarettes.

^g^K6 scores were grouped into five levels of distress (0, 1 to 2, 3 to 5, 6 to 10, and ≥11)

CI = confidence intervals; NH = non-Hispanic; K6 = Kessler 6;aOR = adjusted odds ratio; FPL = federal poverty line

*p<0.05

**p<0.001

## Discussion

This is the first study to investigate the association between level of psychological distress and e-cigarette and cigarette use using data from the 2014 NHIS, which includes the first estimates of e-cigarette usage among US adults. We found that after adjustment for multiple possible confounders, as individuals’ levels of psychological distress increased, they were more likely to have tried e-cigarettes, consistent with the substantial literature concerning cigarette smoking.[30, 36, 37] Moreover, as psychological distress increased, so did rates of current use of cigarettes, dual use and being a former cigarette smoker who had tried e-cigarettes.

Psychological distress is a widely used term that encompasses a number of related psychological phenomena. It sometimes is used to denote the distress that accompanies transient or longstanding stress, including that which frequently accompanies physical health conditions, both acute and chronic.[38–41] It also is an intrinsic component of many mental health problems as well as frequently being used as a general indicator of mental health problems.[42] Psychological distress also has been found to be associated with an increased risk of mortality from several different causes in a dose response pattern.[43–45]

Increased rates of cigarette smoking are well recognized to be highly associated with psychological distress, again in a dose effect pattern.[30, 36, 37] Some of the studies demonstrating this association have used the Kessler scale as the one used in the current study, and one that used this scale also relied on data from the NHIS.[30, 37, 46–52] A recent systematic review of longitudinal studies assessing the relationship between smoking and depression and/or anxiety, however, reported inconsistent findings regarding the direction of this association. [53, 54]

As mentioned in the introduction, it is hoped that e-cigarettes will serve as a useful cessation aid for the general population of cigarette smokers.[55] Individuals with psychological distress have been shown to have more difficulty with smoking cessation[46, 48] than smokers without such distress, with traditional nicotine replacement therapies being less effective for this population,[56] and it has been speculated that e-cigarettes could be a potential aid for smoking cessation for those with mental health problems.[57, 58]

However, in the other population-based study related to this topic, Cummins et al. (2014)[59] found that individuals with mental health problems were more likely to use e-cigarettes than those without such conditions (14.8% vs. 6.6%, respectively), consistent with our findings. Additionally, this study also reported that current smokers with mental health conditions (MHC) were more susceptible to the use of e-cigarettes compared with never-smokers, long-term former smokers, and recent former smokers, suggesting that smokers with MHC have a tendency to perceive e-cigarette use as less harmful and as a quitting aid. Also, a review of clinical trials reported with individuals with severe mental health problems report similar results.[22] This may be reflected in our finding that increasing levels of psychological distress were associated with increasing rates of e-cigarette use among former smokers. Psychologically distressed former smokers may be more likely to use e-cigarettes than non-users, suggesting that e-cigarette use can act as a substitute for cigarette smoking among former smokers with higher levels of psychological distress. A handful of studies have findings that suggest that individuals with mental illness may find e-cigarettes an appealing substitute for combustible cigarettes.[21, 60]

Like prior studies[8, 61], the current one demonstrated that younger adults were more likely to use e-cigarettes, whereas the prevalence of cigarette smoking was higher among older individuals. Those aged 18–34 years with higher levels of psychological distress were more likely to have used e-cigarettes in the past 30 days, despite having not previously smoked cigarettes. Although e-cigarette use might contribute as an aid for smoking cessation or reduction among current smokers[55], question still exists about whether use of e-cigarettes among younger adults may act as a gateway to nicotine addiction, which can trigger cigarette smoking in later life [61–63]. Thus, it might be premature to recommend e-cigarettes as an aid to smoking cessation among younger individuals.

As relates to increased rates of smoking among those with mental health problems, the direction of causality is still unknown, and studies have found that smoking frequently both precedes mental illness, and also arises after the onset of mental illness.[53] While it has been shown that smoking cessation decreases the risk of depression, anxiety, and stress, it is unclear whether other life events led to a decrease in both smoking and mental illness symptoms.[64] It is entirely possible that smoking and poor mental health share a bi-directional relationship [54] and this well may apply to the finding that e-cigarette use increases with mental health problems and increasing psychological distress.

The limitations of this study should be noted. We were unable to establish the causality and temporality of the relationship between psychological distress and e-cigarette and cigarette use due to the nature of this cross-sectional study. Psychological distress was measured based on self-reporting rather than diagnosis by clinicians, but the Kessler 6 has been widely used and validated as a measure of psychological distress, and mental health problems[24, 65] although it does not enable one to know the nature of the mental health problem, or whether the distress is transient or longstanding or related to a trigger such as a stressful life event.[66] Also, the results relied on self-reported information regarding e-cigarette use, which is an approach that has not been validated. Finally, given the NHIS response rate of 58.9%, it is possible that the results were affected by a nonresponse bias.

Future longitudinal studies should clarify whether psychologically distressed individuals are at increased risk of e-cigarette use and/or dual use or whether use of e-cigarettes or both e-cigarettes and cigarettes increases psychological distress. Additionally, it is important to investigate whether e-cigarette use, as a nicotine therapy, has a negative or a positive impact on mental health later in life.

## Ethics statement

This study was exempt from International Review Board (IRB) review because we used a publicly available, unidentifiable dataset. Any of the authors of this manuscript are affiliated with the National Health Interview Survey.

## Conclusions

This study, using data from the NHIS, found that individuals with higher levels of psychological distress were more likely to use not only cigarettes, but also e-cigarettes, compared with non-users. Given the rapid increase in e-cigarette use, especially in younger persons, these results suggest that it is important to recognize use of this agent and its independent association with psychological distress. Moreover, clinicians should be vigilant for mental health problems in their patients who use e-cigarettes.
